# Osteoprotegerin is strongly associated with calcium and magnesium in seminal fluid but not affected by denosumab

**DOI:** 10.1007/s40618-026-02996-z

**Published:** 2026-07-24

**Authors:** Sam Kafai Yahyavi, Mads Joon Jorsal, Emil Brink Wriedt, Niels Jørgensen, Anders Juul, Rune Holt, Martin Blomberg Jensen

**Affiliations:** 1 Division of Translational Endocrinology, Department of Endocrinology and Internal Medicine, Copenhagen University Hospital - Herlev and Gentofte, Herlev, Denmark; 2 Department of Obstetrics and Gynecology, Copenhagen University Hospital - Herlev and Gentofte, Herlev, Denmark; 3 Department of Growth and Reproduction, Copenhagen University Hospital – Rigshospitalet, Copenhagen, Denmark; 4 International Centre for Research and Research Training in Endocrine Disruption of Male Reproduction and Child Health (EDMaRC), Copenhagen University Hospital – Rigshospitalet, Copenhagen, Denmark; 5 Department of Clinical Medicine, Copenhagen University Hospital, Copenhagen, Denmark

**Keywords:** Seminal OPG, sRANKL, Infertility, Minerals, Randomized controlled trial

## Abstract

**Objective:**

While the effect of the RANKL-inhibitor denosumab on serum minerals is well-documented, its impact on seminal fluid minerals is poorly understood. This study examines the correlation between the high levels of soluble-RANKL, osteoprotegerin (OPG), and minerals in seminal fluid and whether this can be influenced by denosumab.

**Methods:**

Two cohorts comprised this study: 100 young men from the general Danish population with no reported fertility issues, and 91 infertile men who were treated with a single dose of denosumab (60 mg), or placebo in a randomized controlled trial assessing the effect on semen quality. Each man underwent a physical examination and semen analysis, including analysis of soluble RANKL, OPG, calcium, magnesium, and phosphate in the seminal fluid.

**Results:**

In both men from the general population and infertile men, seminal OPG concentrations were positively correlated with seminal calcium (r_normal_=0.54 and r_infertile_=0.81), seminal magnesium (r_normal_=0.58 and r_infertile_=0.75), and a weak negative correlation was seen with seminal phosphate (r_normal_=-0.17 and r_infertile_=-0.34) concentrations. Seminal soluble RANKL was not associated with seminal minerals. Following 60 mg denosumab treatment, no changes were observed after 160 days in seminal calcium (*p* = 0.42), magnesium (*p* = 0.55), or phosphate concentrations (*p* = 0.63). Changes in seminal OPG were moderately negatively correlated with changes in serum AMH (*r*=-0.32), LH (*r*=-0.30), and FSH (*r*=-0.31).

**Conclusion:**

A strong correlation between OPG and minerals in seminal fluids from both normal and infertile men suggests that the release of these factors by the prostate or seminal vesicle is regulated by the same factors, but cannot be modified by a short-term systemic RANKL inhibition with denosumab.

**Clinical trial registration:**

ClinicalTrials.gov NCT03030196. Registered January 24, 2017.

## Introduction

The relationship between osteoprotegerin (OPG) and receptor activator of nuclear factor kappa-Β ligand (RANKL) is critical for bone metabolism, where treatment with denosumab, a RANKL inhibitor, is a widely used treatment for osteoporosis [1, 2]. However, emerging evidence suggests their involvement in male reproductive physiology [3], and both OPG and RANKL have been identified in seminal fluid, where their functional significance remains under investigation [4]. There is a need to explore new regulators of fertility beyond the traditional hormonal pathways, as the declining fertility rates necessitate an increased focus on etiology and novel treatment options for male infertility [5].

In the testes, Sertoli cells are strongly influenced by endocrine signals, particularly follicle-stimulating hormone (FSH) and testosterone [6]. FSH stimulates Sertoli cells to promote sperm production and the release of various factors, such as anti-Müllerian hormone (AMH) and inhibin B, which have been suggested to be clinical markers of Sertoli cell function and spermatogenesis [7, 8]. Sertoli cells also express RANKL, which signals through its receptor, RANK, expressed in spermatogonia and spermatocytes [3]. Previous translational studies have shown that serum AMH could serve as a marker for preserved Sertoli cell function and thereby be a serum biomarker for selecting infertile men, who are more likely to benefit from stimulatory treatments with denosumab [4, 9]. However, randomized controlled trials have recently shown that the response to treatment with denosumab varies greatly, with some men benefiting while others experience no changes, and few even have detrimental effects [3, 4]. These conflicting results show that other effects besides Sertoli-germ cell interaction may occur, such as the influence of systemic and local mineral homeostasis, which may influence reproductive function. Clinical experience with denosumab largely derives from elderly populations, while evidence in younger individuals remains limited. Younger patients may exhibit distinct biological responses, potentially influencing seminal fluid parameters and reproductive outcomes.

While the effect of denosumab treatment on serum mineral concentrations is well-documented [10], its impact on seminal fluid composition is more complex and remains poorly understood. Seminal fluid contains a mix of proteins, enzymes, minerals, and various other factors. Animal studies have shown that spermatozoa experience significant fluctuations in concentrations of calcium, magnesium, phosphate as they move from the testes through the male reproductive tract [11, 12]. Minerals are particularly essential for spermatogenesis, and in the epididymis for inducing sperm motility and subsequently to maintain sperm quiescence during storage while they await ejaculation. Calcium acts as a key intracellular messenger in sperm, directly regulating motility and the acrosome reaction [13], while magnesium is necessary for ATP production and is involved in sperm motility and capacitation processes [14]. After ejaculation, the fluid buffers the sperm upon arrival in the vagina, where sperm survival is crucial until they encounter the oocyte, while also enabling capacitation and the acrosome reaction [15]. If the regulation of minerals can be modulated in the testes, epididymis, prostate, or seminal vesicle, it could lead to targeted strategies in treating male infertility and specific diseases in these organs. It has previously been shown that RANKL, RANK, and OPG are expressed in the epididymis, prostate, and seminal vesicle [3], and therefore, this study aims to investigate the relationship between the RANKL/OPG system and mineral concentrations of calcium, phosphate, and magnesium in seminal fluid and to determine whether these can be altered by denosumab treatment.

## Materials and methods

### Study population

This study comprised two separate study cohorts, and both studies were carried out at the Department of Growth and Reproduction, Copenhagen University Hospital, Rigshospitalet, Copenhagen (Denmark). (1) The cohort of 100 men was from a general Danish population study of the semen quality of young men [16–18]. The men were recruited at a compulsory evaluation for military service and were at this stage invited to participate in the study between January and December 2018, irrespective of their qualification for military service. In the study, each participant provided a semen and a blood sample and underwent a physical examination. The study approval was obtained from the local ethical committee (journal no: H-KF-289428). All participants returned written informed consent prior to participation in the study. (2) The data from the 91 infertile men stems from “Copenhagen First”, a randomized controlled trial designed to investigate the effect of a single dose of denosumab (60 mg) vs. placebo (saline) on semen quality [4]. The study was a randomized controlled trial conducted between 2017 and 2020. The main results of the randomized clinical trial including details on the sample size calculations have been published recently [4]. Eligible participants were men (age 18 to 65 years) from infertile couples whose semen analysis showed impaired semen quality (Sperm concentration < 15 million/ml, or < 40% of motile permatozoa, or < 4% morphologially normal spermatozoa) and a minimum sperm concentration > 0.05 million/mL, while exclusion criteria included chronic diseases, previous malignancies, and disturbances in the bone metabolism (i.e., severe vitamin D or calcium deficiencies or osteoporosis) [4]. The infertile cohort was otherwise generally healthy apart from impaired semen quality, as men with known endocrine or genetic disorders were excluded. Within the cohort, 54% had a clinical diagnosis of varicocele (13% grade 3), 5% had a history of cryptorchidism, and approximately 10% were smokers. Each participant provided semen samples at three time points (day 1, day 80, and day 160) for routine analysis, and these have been analyzed for the content of seminal calcium, magnesium, and phosphate. Additionally, all participants received daily supplementation with 400 mg of calcium and 25 µg of cholecalciferol from the start of treatment through follow-up (160 days) to minimize the risk of hypocalcemia and ensure treatment safety.

### Biochemical and semen analysis

Semen samples were collected by masturbation. Duration of abstinence, fever, and spillage were self-reported. Analysis of semen samples has previously been described in detail [3, 4] In seminal fluid and serum, total calcium (intra-assay coefficient of variation (CV) 2.5%), phosphate (CV < 3%), and magnesium (CV < 6%) were measured using Cobas 8000. Serum and seminal sRANKL (CV 12%) were measured by ELISA (#BI-20462, Biomedica, Austria), while serum and seminal OPG (CV 5%) were measured by ELISA (#BI-20403, Biomedica, Austria). Serum LH (CV 6%) and FSH (CV 4%) were measured using time-resolved immunofluorometric assay Delfia (Wallac, Finland), Inhibin B measurements (CV 11%) were performed by two-sided ELISA inhibin B gen II (A81301, Beckman Coulter, US). AMH (CV 8%) was analyzed by ELISA (#A79765, Beckman Coulter, US). Serum estradiol (CV 12%) and serum testosterone (CV 10%) concentrations were measured by enzyme-linked immunoassay (Access 2, Beckman Coulter). Free testosterone was calculated using the Vermeulen formula.

### Statistical analysis

**Fig. 1:**
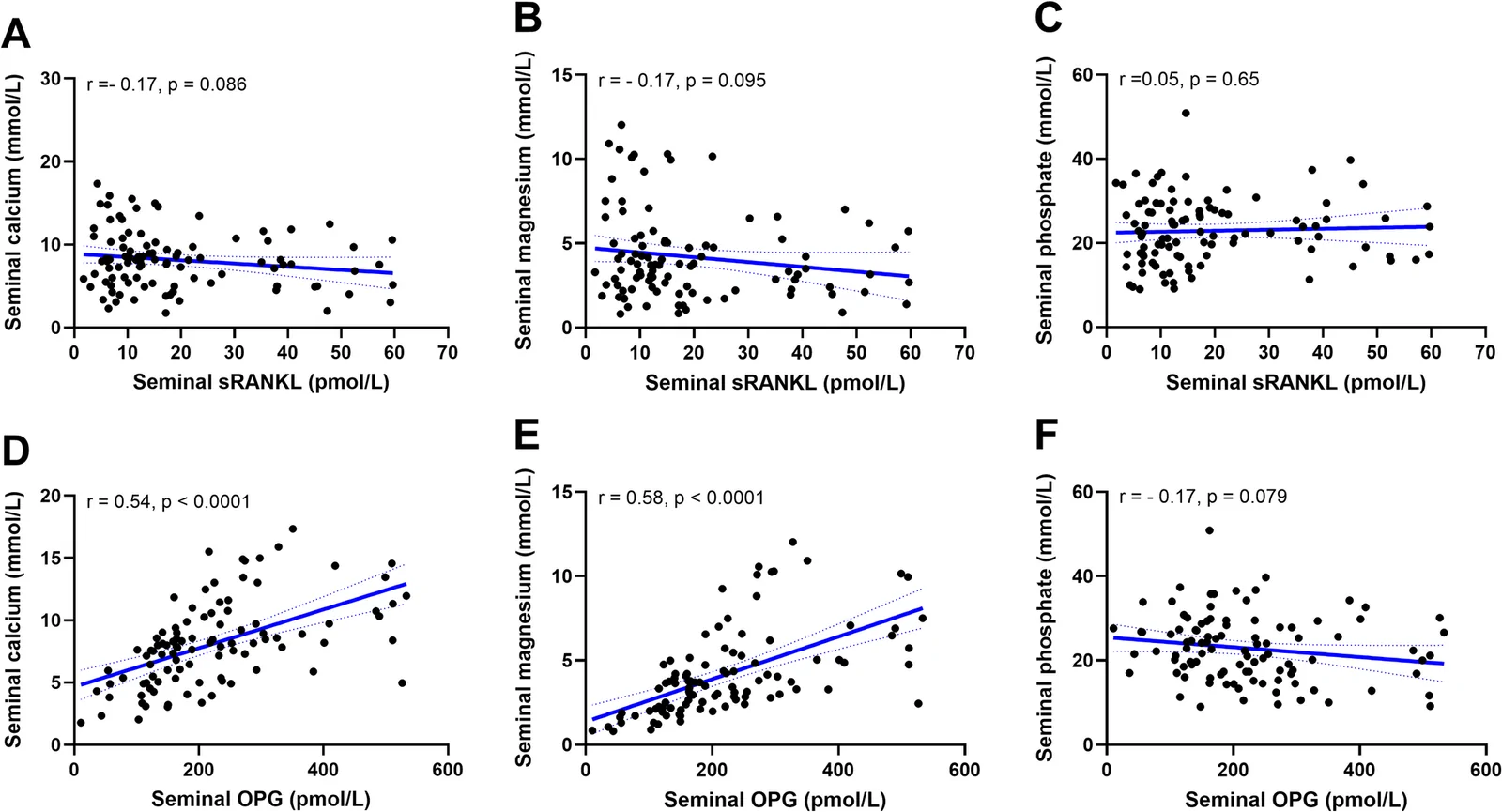
Correlations between seminal calcium, magnesium, and phosphate concentrations with seminal sRANKL and OPG concentrations in 100 men from the general population. Seminal concentrations of sRANKL and OPG and correlations with seminal calcium, magnesium, and phosphate concentrations in 100 men from the general population. (**A**) Seminal sRANKL and seminal fluid calcium concentration, (**B**) Seminal sRANKL and seminal fluid magnesium concentration, (**C**) Seminal sRANKL and seminal fluid phosphate concentration, (**D**) Seminal OPG and seminal fluid calcium concentration, (**E**) Seminal OPG and seminal fluid magnesium concentration, (**F**) Seminal OPG and seminal fluid phosphate concentration. Each dot represents an individual, and the blue line shows regression line with 95% confidence intervals. Pearson’s correlation coefficient and p-value are shown

**Fig. 2:**
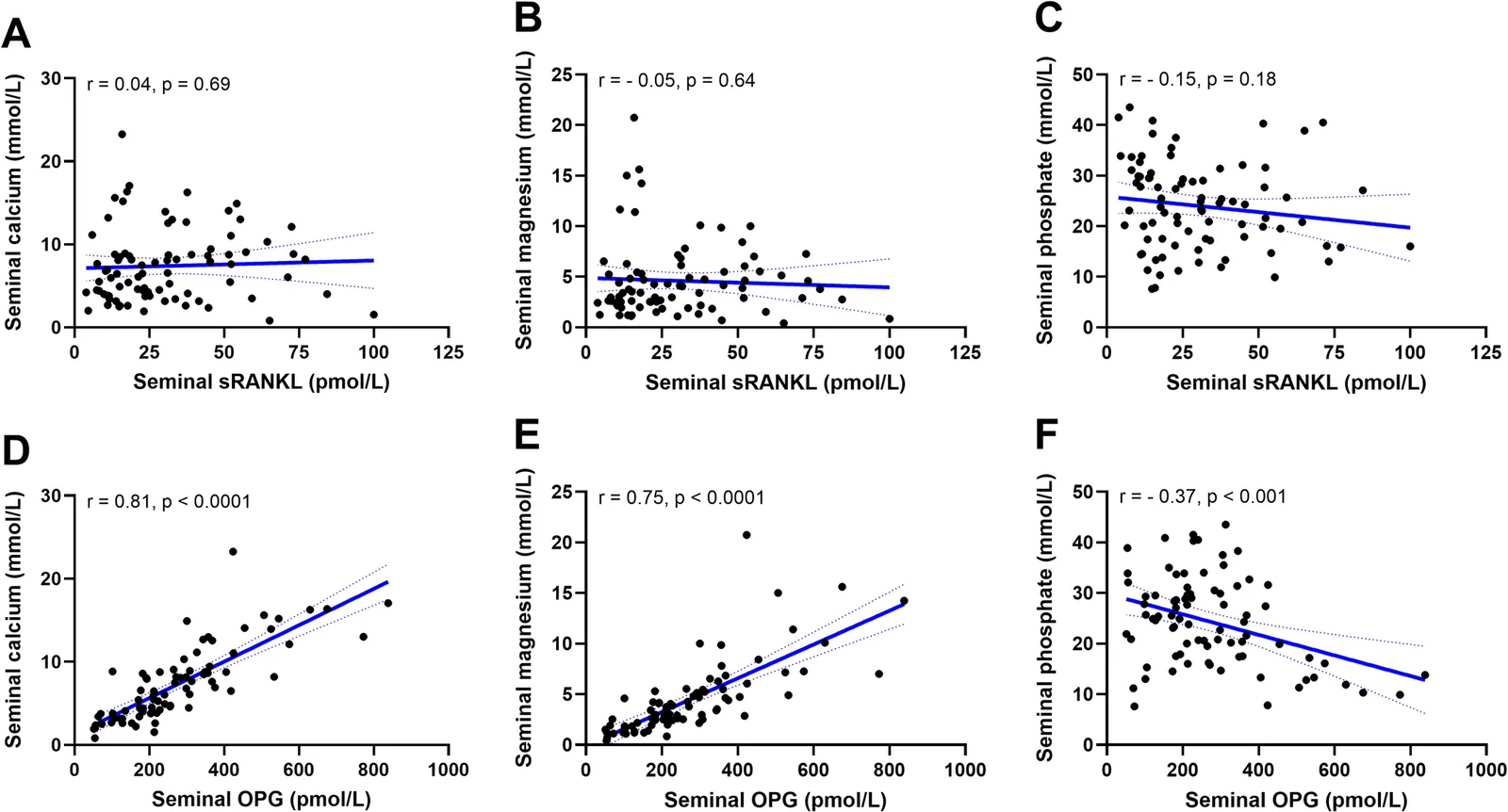
Correlations between seminal calcium, magnesium, and phosphate concentrations with seminal sRANKL and OPG concentrations in 91 infertile men. Seminal concentrations of sRANKL and OPG and correlations with seminal calcium, magnesium, and phosphate concentrations in 91 infertile men. (**A**) Seminal sRANKL and seminal fluid calcium concentration, (**B**) Seminal sRANKL and seminal fluid magnesium concentration, (**C**) Seminal sRANKL and seminal fluid phosphate concentration, (**D**) Seminal OPG and seminal fluid calcium concentration, (**E**) Seminal OPG and seminal fluid magnesium concentration, (**F**) Seminal OPG and seminal fluid phosphate concentration. Each dot represents an individual, and the blue line shows regression line with 95% confidence intervals. Pearson’s correlation coefficient and p-value are shown

**Fig. 3:**
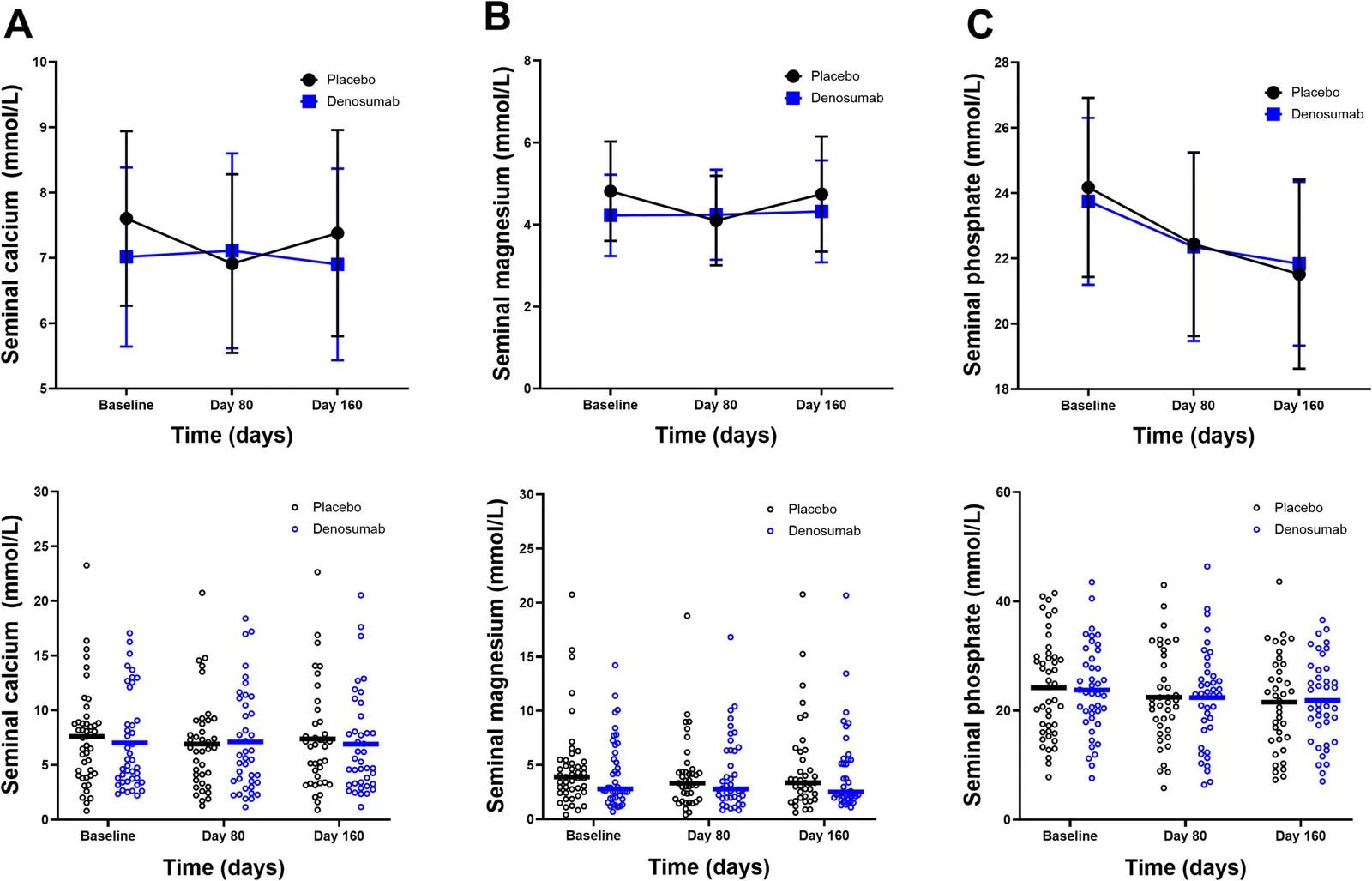
Concentrations of seminal calcium, magnesium, and phosphate at baseline, and 80 and 160 days after treatment with 60 mg denosumab s.c. compared to placebo. Concentrations of seminal calcium, magnesium, and phosphate at baseline and after 80 and 160 days after treatment with 60 mg denosumab compared to placebo (**A**) Seminal calcium, (**B**) Seminal magnesium, (**C**) Seminal phosphate. In the top figures, dots represent the median with IQR for denosumab and placebo, respectively while in the bottom figures, dots represent an individual and lines median

In the RCT, analyses were conducted according to the intention-to-treat principle. Participants contributed data at all assessed time points (baseline, day 80, and day 160). In Tables 1 and 2, variables are presented as medians with interquartile range [IQR] for continuous variables. Categorical variables were presented as numbers with percentages. In Figs. 1 and 2, correlations between variables or changes in variables were assessed with Pearson’s correlation coefficient after normality was verified. To assess the potential regulators of seminal OPG, the correlations between changes in seminal OPG and various serum and seminal variables were also assessed with Pearson’s correlation coefficient (Table 3). Given the exploratory nature of these analyses, no correction for multiple testing was applied. The differences between the randomized groups were tested with Mann Whitney U test (Fig. 3). Observations with missing data were excluded from the analyses. A p-value below 0.05 was considered statistically significant. All statistical calculations were conducted using the statistical software R, version 3.4.1 (R Core Team 2021. R: A Language and Environment for Statistical Computing. R Foundation for Statistical Computing, Vienna, Austria. http://www.R-project.org).

**Table 1:** Baseline characteristics of the 100 men from the general population and 91 infertile men included in the study

Characteristics	Men from the GP (*n* = 100) Median [IQR]	Infertile men (*n* = 91) Median [IQR]	*P*-value
Age (years)	19 [19, 19]	33 [30, 38]	**< 0.001**
BMI (kg/m^2^)	22.0 [20.1, 23.6]	25.9 [23.6, 28.2]	**< 0.001**
FSH (IU/L)	2.9 [2.0, 4.1]	4.8 [3.8, 7.6]	**< 0.001**
LH (IU/L)	2.8 [2.2, 3.9]	4.1 [2.7, 5.1]	**< 0.001**
SHBG (nmol/L)	32 [25, 40]	32 [23, 47]	0.12
Inhibin B (pg/mL)	176 [136, 225]	115 [85, 160]	**< 0.001**
Total testosterone (nmol/L)	19.9 [17.2, 22.8]	16.3 [13.2, 23.2]	**0.048**
Free testosterone (pmol/L)	435 [380, 523]	364 [287, 450]	**< 0.001**
Estradiol (pmol/L)	47.0 [34.0, 67.5]	73.4 [55.7, 87.2]	**< 0.001**
Testosterone/estradiol ratio	430 [310, 560]	251 [201, 308]	**< 0.001**
Seminal sRANKL (pmol/L)	13.3 [8.5, 23.4]	24.6 [14.9, 42.9]	**< 0.001**
Seminal OPG (pmol/L)	200.9 [140.9, 273.9]	240.9 [176.2, 356.7]	**0.014**
Seminal calcium (mmol/L)	8.0 [5.4, 10.0]	6.6 [4.0, 8.9]	0.16
Seminal magnesium (mmol/L)	3.6 [2.4, 5.1]	3.5 [2.5, 5.5]	0.47
Seminal phosphate (mmol/L)	22.4 [17.0, 27.8]	23.8 [17.3, 29.7]	0.38

Data is presented as median (IQR). Abbreviations: GP – General population, SHBG - Sex hormone-binding globulin, FSH - Follicle-stimulating hormone, LH - Luteinizing hormone, OPG - osteoprogeterin

**Table 2:** Infertility history and semen variables of the 100 men from the general population and 91 infertile men included in the study

Characteristics	Men from the GP (*n* = 100) Median [IQR]	Infertile men (*n* = 91) Median [IQR]	*P*-value
Duration of infertility (months)	N/A	20 [15, 27]	N/A
Varicocele, no, (%)	1 (1%)	52 (57.1%)	**< 0.001**
Cryptorchidism, no, (%)	6 (6%)	5 (5.5%)	0.81
Mean testis size, ultrasound (mL)	13.1 [10.8, 15.7]	11.0 [9.2, 14.0]	**< 0.001**
Duration of abstinence (days)	2.5 [2.0, 3.0]	3.5 [3.0, 4.0]	**< 0.001**
Semen volume (mL)	3.0 [2.3, 4.0]	3.8 [2.9, 4.7]	**< 0.001**
Sperm concentration (10^6^/mL)	44.5 [25.3, 70.0]	7.2 [3.3, 12.5]	**< 0.001**
Total sperm count (10^6^)	133 [68, 204]	24 [10, 49]	**< 0.001**
Motile spermatozoa (ABC%)	78 [69, 82]	44 [31, 57]	**< 0.001**
Total no. of motile spermatozoa (10^6^)	101 [48, 161]	11 [3, 26]	**< 0.001**
Progressively motile spermatozoa (AB%)	72 [64, 77]	32 [20, 44]	**< 0.001**
Total no. of prog. motile spermatozoa (10^6^)	89 [44, 152]	8 [2, 20]	**< 0.001**
Normal sperm morphology (%)	7.0 [4.5, 10.0]	2.0 [1.0, 3.3]	**< 0.001**
Total no. of morph. normal spermatozoa (10^6^)	10.0 [2.9, 16.8]	0.4 [0.1, 1.2]	**< 0.001**

Data is presented as median (IQR) unless otherwise indicated

**Table 3:** Correlations between changes in seminal OPG and various variables from baseline to day 80 in the 91 infertile men participating in the RCT

	∆ Seminal OPG	
	All participants	Placebo only
∆ Albumin	-0.05	0.02
∆ Serum total calcium	-0.15	-0.14
∆ Serum ionized calcium	-0.01	0.13
∆ Serum phosphate	0.10	0.00
∆ Serum alk. phosphatase	0.09	0.01
∆ Serum PTH	0.00	-0.08
∆ Serum prolactin	-0.01	0.26
∆ Serum 25(OH)D	-0.02	-0.03
∆ Serum LH	-0.14	**-0.30**
∆ Serum FSH	-0.10	-**0.31**
∆ Serum Inhibin B	-0.19	-0.16
∆ Serum AMH	**-0.32**	**-0.32**
∆ Serum testosterone	-0.16	0.01
∆ Serum estradiol	-0.20	-0.06
∆ Serum SHBG	-0.16	0.11
∆ Serum OPG	-0.01	0.09

Data presented as correlation coefficients between changes in seminal OPG and changes in the different variables on day 80. Bold text marks a correlation above 0.30 considered moderate. Analysis performed with Pearson’s correlation test

## Results

### Baseline characteristics

A total of 100 young men (median age 19 years) from the general Danish population and 91 infertile men (median age 33 years) were included in the study. While the young men cannot be characterized as healthy as they were chosen randomly, they have healthier characteristics, i.e., BMI at 22.0 kg/m² compared to the infertile men (25.9 kg/m²). Reproductive hormone concentrations also differed as expected, as the infertile men had lower testosterone concentrations, while LH and FSH concentrations were higher with lower inhibin B (Table 1). The median duration of infertility was just under 2 years, and the infertile men had markedly impaired semen parameters, with a sperm concentration of 7.2 million/mL, a total sperm count of 24 million, and 44% motile spermatozoa. In contrast, men from the general population had substantially higher semen quality, with a sperm concentration of 44.5 million/mL, a total sperm count of 133 million, and 78% motile spermatozoa (all parameters shown in Table 2). The infertile men had higher concentrations of both seminal soluble RANKL (sRANKL) and OPG, and lower concentrations of seminal calcium compared to the young men. Seminal magnesium and phosphate concentrations were comparable (Table 1).

### Association of seminal sRANKL and OPG with calcium, magnesium, and phosphate

In young men from the general population, seminal sRANKL concentrations showed no significant correlation with seminal fluid calcium (*r* = -0.17, *p* = 0.086; Fig. 1A), magnesium (*r* = -0.17, *p* = 0.095; Fig. 1B), or phosphate concentrations (*r* = 0.05, *p* = 0.65; Fig. 1C). In contrast, seminal OPG concentrations were positively correlated with both seminal calcium (*r* = 0.54, *p* < 0.0001; Fig. 1D) and magnesium levels (*r* = 0.58, *p* < 0.0001; Fig. 1E). No correlation was found between seminal OPG and phosphate concentrations (*r* = − 0.17, *p* = 0.079; Fig. 1F). Similarly, in the infertile men, seminal sRANKL concentrations were not significantly correlated with calcium (*r* = 0.04, *p* = 0.69; Fig. 2A), magnesium (*r* = − 0.05, *p* = 0.64; Fig. 2B), or phosphate levels (*r* = 0.15, *p* = 0.18; Fig. 2C). Here, seminal OPG concentrations were strongly positively associated with both seminal calcium (*r* = 0.81, *p* < 0.0001; Fig. 2D) and magnesium (*r* = 0.75, *p* < 0.0001; Fig. 2E), while a moderate negative correlation was observed between seminal OPG and phosphate concentrations (*r* = − 0.45, *p* = 0.001; Fig. 2F).

### Changes in seminal calcium, magnesium, and phosphate after treatment with denosumab

In the RCT, 43 men were treated with denosumab while 48 received placebo. The men in the intervention group experienced no change in seminal calcium, magnesium, or phosphate concentrations over the 80 and 160-day period (Fig. 3). Median seminal calcium remained stable in the denosumab treated group (7.0, 7.1, and 6.9 mmol/L at baseline, day 80, and day 160, respectively), while the placebo group experienced a slight drop from 7.6 mmol/L at baseline to 6.9 mmol/L on day 80, before it returned to 7.4 mmol/L at day 160 (*p* = 0.42). Similarly, the placebo group saw a slight drop in median magnesium concentrations at day 80 (4.8, 4.1, and 4.7 mmol/L at baseline, day 80, and day 160, respectively), while the denosumab treated group had stable concentrations (4.2, 4.2, and 4.3 mmol/L), but with no differences between groups (*p* = 0.55). Phosphate concentrations decreased in both denosumab and placebo groups at day 80 and day 160 compared to baseline, with no differences between the two groups (*p* = 0.63). The effect of denosumab treatment on serum and seminal RANKL and OPG has been published earlier [4]. In brief, a suppression of sRANKL in the seminal fluid was seen as men treated with denosumab experienced a decrease in concentrations at day 80 compared with placebo group (1.5 vs. 25 pmol/L, *p* < 0.001). There was no difference in seminal fluid OPG concentrations (188 vs. 237 pmol/L, *p* = 0.21). There was a drop in both serum RANKL and serum OPG in the denosumab group, but it was only serum RANKL that was significant when compared to the changes in the placebo group (0.07 vs. 0.20 pmol/L, *p* < 0.001).

### Correlations of changes in seminal OPG with other variables

To assess the potential regulators of seminal OPG, the correlations between changes in seminal OPG and various serum and seminal variables from baseline to day 80 are shown in Table 3. Among all participants, most correlations were weak and not clinically meaningful. The only moderate correlation observed was a negative association between changes in seminal OPG and serum AMH (*r* = − 0.32, *p* < 0.001), suggesting that an increase in seminal OPG was associated with a decrease in serum AMH levels. Exploiting the RCT design and focusing on only the placebo group to leave out a possible effect of denosumab treatment, similar patterns were observed. Notably, moderate negative correlations were also seen between changes in seminal OPG and serum LH (*r* = − 0.30, *p* < 0.001) as well as serum FSH (*r* = − 0.31, *p* < 0.001). Additionally, the correlation with serum AMH remained moderate and negative (*r* = − 0.32, *p* < 0.001). All other variables, including serum calcium, phosphate, reproductive hormones, and serum 25OHD, showed only weak or negligible associations with changes in seminal OPG.

## Discussion

This study shows that seminal OPG concentrations are positively associated with seminal calcium and magnesium in both young men from the general population and infertile men. While seminal sRANKL concentrations did not correlate with any of the measured minerals, OPG consistently showed strong positive associations with calcium and magnesium across both groups of men, suggesting that OPG may play a role in regulating mineral homeostasis in the seminal fluid independently of sRANKL. The observed strong correlation between seminal OPG and calcium and magnesium does suggest a physiological link between OPG and mineral homeostasis within the male reproductive tract. OPG regulates bone resorption and thus inhibits the release of minerals from the skeleton through its inhibition of RANKL [2], but it has also, in recent years, been increasingly recognized for its broader biological roles, including its presence and potential functions in non-skeletal tissues such as the testes and accessory glands [3, 4]. However, despite these strong correlations, a single-dose administration of denosumab did not affect the concentrations of calcium, phosphate, or magnesium in seminal fluid. This implies that mineral content in seminal fluid is not directly linked to systemic changes in mineral status but is controlled by other yet unidentified regulators. This aligns with existing knowledge of the unique microenvironment of the male reproductive tract, from which the seminal fluid is comprised of different organs, including the testes, epididymis, seminal vesicles, and prostate [19]. Also, the local environment includes tight junctions, selective permeability, and specialized epithelial transporters [20] that maintain a distinct composition and biochemical milieu. Mineral metabolism in seminal fluid is a tightly regulated process that plays a crucial role in sperm function, including motility, capacitation, and acrosome reaction [21]. It is increasingly recognized as an important determinant of male reproductive function. Seminal plasma contains tightly regulated concentrations of calcium, phosphate, zinc, and magnesium [22]. The prostate and seminal vesicles contribute significantly to this ionic composition through active secretion and enzymatic regulation [23, 24]. Calcium signaling, in particular, is critical for sperm activation and the acrosome reaction, while phosphate balance may influence energy metabolism. Higher seminal calcium has been associated with sperm concentration [25], and lower seminal magnesium is linked to reduced sperm motility and concentration [22]. Zinc is abundant in seminal plasma and has antioxidant and structural roles. Dysregulation of these minerals has been associated with impaired sperm quality and fertility outcomes, and seminal phosphate levels may be influenced by factors such as testosterone [26].

In such a context, proteins like OPG may serve paracrine or autocrine functions that differ from their systemic roles as known, i.e., tumor environments [27, 28]. The lack of correlation between seminal sRANKL and any of the assessed minerals further supports the notion that OPG may have an independent regulatory role, rather than simply acting as a decoy receptor for RANKL or as a regulator of apoptosis through tumor necrosis factor (TNF)-related apoptosis-inducing ligand (TRAIL) [29]. This decoupling raises interesting hypotheses about the non-classical roles of OPG or context-dependent expression and activity of RANKL. This suggestion is also supported by showing that denosumab lowered seminal fluid soluble RANKL but had no effect on seminal OPG levels [4], which shows that OPG is controlled by another yet unidentified factor. This apparent disconnect between correlation and causation between seminal OPG and minerals warrants further exploration. Most of the data on the effect of denosumab on bone health originates mainly from post-menopausal women with osteoporosis or men with prostate cancer. Therefore, more studies of denosumab treatment in younger men are needed in order to evaluate the effect on male reproductive organ function. The infertile men in our study had higher gonadotropins (LH and FSH) and lower testosterone compared to the young men from the general population. Inhibin B levels were also lower in the infertile men, further reflecting impaired testicular function. These hormonal differences align with the expected hormonal profiles typically observed in male infertility. Interestingly, seminal fluid OPG was only negatively associated with seminal fluid phosphate in infertile men and not men from the general population, which highlights a potential difference that may be of relevance. However, this difference can also be due to a 20% higher concentration of seminal OPG seen in infertile men compared to men from the general population, including a higher upper range of values, which highlights the correlation. Since both OPG and sRANKL concentrations were higher in infertile men compared to healthy young men, it may reflect altered regulation of the RANKL/OPG signaling system within the reproductive tract. Increased OPG and sRANKL may reflect a potential compensatory response to disturbances in tissue remodelling, inflammation, or oxidative stress. As both OPG and RANKL are involved in immune signalling pathways, their altered levels may also suggest possible local immune activation. It may also result from altered permeability or transport between blood and reproductive tissues, allowing higher levels to accumulate locally. The correlation between OPG and calcium/magnesium does suggest that dysregulated mineral balance in infertile men could contribute to the observed elevations. The interventional data showed that treatment with denosumab did not affect seminal calcium, magnesium, or phosphate levels evaluated after 80 or 160 days. These findings imply that systemic inhibition of RANKL is not sufficient to alter the mineral composition of seminal fluid in the short term, further supporting the hypothesis that local regulatory mechanisms, rather than systemic mineral metabolism, predominate in the control of seminal mineral concentrations. Another explanation of the disconnect between denosumab treatment and seminal mineral concentrations could be that OPG and seminal minerals are regulated by, for instance, androgens. We observed a few moderate inverse correlations between changes in seminal OPG and serum AMH, LH, and FSH. While the clinical significance of these associations remains unclear, they may point toward a role for reproductive hormones either directly or indirectly regulating OPG and maybe mineral homeostasis.

A key strength in this study is the integration of data from two different cohorts. One of infertile men and one with data from young men from the general Danish population, which makes a strong case for the correlations between seminal OPG and minerals. By exploiting the RCT design, we were able to assess whether systemic denosumab treatment led to changes in seminal mineral concentration by directly comparing outcomes between the treatment and placebo groups. Ideally, a larger cohort would have increased the power and generalizability of our findings, however, recruiting just under 100 men for a randomized controlled trial in the context of male infertility is considered a strong achievement, given the well-documented challenges associated with enrollment in this population [30]. Despite these strengths, several limitations must be acknowledged. The data from men in the general population were only cross-sectional, which prevents us from concluding temporal changes or causal relationships. The follow-up duration, while sufficient to detect changes, may not have been long enough to evaluate the long-term effects of denosumab on seminal mineral metabolism. As denosumab is not an approved treatment for the indication of male infertility, prolonged or repeated administration could expose them to unnecessary risks without clinical benefit. A single injection allowed us to observe short-term biological effects while minimizing participant risk, however, this design does not provide a complete picture of the long-term impact of denosumab treatment. This is particularly relevant as denosumab’s pharmacokinetics suggest potential rebound effects beyond the follow-up period when assessing its effects on bone markers. A limitation of this study is the lack of multivariable analyses to adjust for confounding factors affecting seminal mineral concentrations, such as age, BMI, hormonal status, and abstinence duration. All participants in the intervention trial also received calcium and vitamin D supplementation, which may have influenced mineral levels and the observed effects of denosumab. Although the randomized, placebo-controlled design helps reduce bias, unadjusted analyses limit interpretation, and residual confounding cannot be excluded. Importantly, this was an exploratory analysis, and therefore multivariable adjustments were not performed. Future studies should use multivariable models to clarify the independent effects of denosumab on seminal mineral homeostasis. Another limitation is also the lack of systematic transrectal ultrasound data, which prevented assessment of correlations between OPG/RANKL levels and structural or functional alterations of the prostate and seminal vesicles. Finally, as the infertile men were recruited from a tertiary center in Copenhagen, the findings may not be fully generalizable to broader populations of men, including those who are undiagnosed or not actively seeking fertility evaluation. While the biological mechanisms studied are likely conserved, our findings are based on a Danish population, and caution is warranted when generalizing to other populations due to potential genetic, environmental, and lifestyle differences.

In conclusion, despite the observed strong correlation between seminal OPG and calcium and magnesium, our findings indicate that a single dose of denosumab does not influence seminal mineral concentrations after a short-term follow-up. This suggests that while production and release of classical bone factors such as OPG may be linked with the seminal mineral content, short-term RANKL inhibition does not appear to alter mineral content in seminal fluid. Further studies should aim to identify the regulators of seminal fluid minerals and OPG.

## Data Availability

The data that support the findings of this study are available from the corresponding author, M.B.J., upon reasonable request.
